# Summary of the National Advisory Committee on Immunization (NACI) Statement—Recommendation on Repeated Seasonal Influenza Vaccination

**DOI:** 10.14745/ccdr.v49i04a02

**Published:** 2023-04-01

**Authors:** Angela Sinilaite, Kelsey Young, Jesse Papenburg

**Affiliations:** 1 Centre for Immunization Readiness, Public Health Agency of Canada, Ottawa, ON; 2 NACI Influenza Working Group Chair; 3 Division of Pediatric Infectious Diseases, Department of Pediatrics, Montréal Children's Hospital of the McGill University Health Centre, Montréal, QC; 4 Division of Microbiology, Department of Clinical Laboratory Medicine, Optilab Montréal - McGill University Health Centre, Montréal, QC; 5 Department of Epidemiology, Biostatistics, and Occupational Health, School of Population and Global Health, McGill University, Montréal, QC

**Keywords:** National Advisory Committee on Immunization, Canada, NACI, influenza, influenza vaccine, guidance, vaccine effectiveness, repeated vaccination

## Abstract

**Background:**

Influenza vaccination is recommended annually; however, some studies have raised questions regarding whether repeated influenza vaccine administration may have unintended negative consequences for seasonal protection.

**Methods:**

The National Advisory Committee on Immunization (NACI) Influenza Working Group undertook an overview of systematic reviews on the effects of repeated influenza vaccination on vaccine effectiveness, efficacy, and immunogenicity. A systematic assessment of programmatic factors was conducted according to established NACI methods. The NACI evidence-based process was used to critically appraise the available evidence and to review recommendations.

**Results:**

The evidence base consisted of four eligible systematic reviews/meta-analyses. Repeated vaccination, including the current season, was consistently more effective than no vaccination in the current season. The evidence showed no significant difference or predictable trend in vaccine efficacy or effectiveness between vaccinations in two consecutive seasons compared to vaccination in the current season only.

**Conclusion:**

Overall, NACI concluded that there is evidence to recommend annual influenza vaccination, irrespective of whether an individual received the seasonal influenza vaccine in previous seasons. It is neither currently feasible nor warranted to modify existing annual influenza vaccination programs to account for potential negative or positive interference. NACI continues to strongly recommend that seasonal influenza vaccine should be offered annually to everyone six months of age and older who does not have contraindications to the vaccine, irrespective of previous seasons’ influenza vaccination status.

## Introduction

The influenza vaccine is a critical tool to protect against influenza-related disease and to reduce the influenza-associated burden on the Canadian healthcare system. Influenza vaccination is repeated annually due to waning immunity and the tendency of influenza viruses to mutate frequently, requiring changes in the vaccine formulation. To reduce the morbidity and mortality associated with influenza, National Advisory Committee on Immunization (NACI) recommends annual influenza vaccination for everyone six months of age and older who does not have contraindications to the vaccine ((1)).

A study published in the 1970s ((2)) raised questions about a potential negative impact of prior influenza vaccination on current season influenza vaccine effectiveness (VE) and conflicting results on whether repeated annual seasonal influenza vaccination could have unintended negative consequences for seasonal protection have also been reported ((3–8)). The degree to which repeated vaccination and other factors (e.g. vaccine strain match to circulating strains, initial exposure to influenza virus and egg-adaptive mutations) affect VE is still not fully understood and varies season to season. Furthermore, the complex interplay of factors affecting an individual's immune response to influenza vaccination makes it extremely difficult to make predictions far enough in advance of the next influenza season to help inform vaccine policy or administration practice changes.

NACI was asked to assess the effects of repeated influenza vaccination on VE, efficacy and immunogenicity with the purpose of evaluating the overall impact of this phenomenon and to provide an evidence base for population-level and individual-level vaccination decisions regarding annual influenza vaccination.

## Methods

The NACI Influenza Working Group undertook an overview of existing systematic reviews according to a written protocol specified *a priori* that included review questions, search strategy, inclusion and exclusion criteria and quality assessment. The following research question and accompanying population, intervention, comparison(s) and outcome(s) (PICO) (**Table 1**) was developed to guide the evidence review: What are the effects of repeated seasonal influenza vaccination on VE, efficacy and immunogenicity?

**Table 1:** Population, intervention, comparator(s), outcome(s) criteria guiding NACI’s evidence review

PICO	Criteria
Population	Adults and children
Intervention	Seasonal influenza vaccination in prior season(s) and current season
Comparison	Seasonal influenza vaccination in prior season(s) only OR in current season only OR unvaccinated in any season included in the study
Outcome	Vaccine efficacy or immunogenicity in the current season
Study design	Systematic review or meta-analysis

Abbreviation: NACI, National Advisory Committee on Immunization

To support this work, a systematic assessment of ethics, equity, feasibility, and acceptability of influenza vaccine guidance was also conducted according to established NACI methods ((9)). The NACI evidence-based process ((10)) was used to assess the available evidence and develop a new recommendation. Full details and results are presented in the NACI *Recommendation on Repeated Seasonal Influenza Vaccination* ((11)).

## Results

The NACI’s evidence base encompassed an overview of four systematic reviews (SRs)/meta-analyses (MAs) ((12–15)) on the effects of repeated influenza vaccination on vaccine efficacy or effectiveness, analyzing findings from a total of 24 unique primary studies. None of the SRs/MAs included primary studies that assessed immunogenicity. Based on the available evidence, NACI issued a new recommendation on repeated seasonal influenza vaccination.

### Recommendation

NACI continues to recommend that seasonal influenza vaccine should be offered annually to everyone six months of age and older who does not have contraindications to the vaccine, irrespective of previous seasons’ influenza vaccination status. (*Strong NACI Recommendation*)

• **NACI concludes that there is fair evidence to recommend annual influenza vaccination, irrespective of whether an individual received the seasonal influenza vaccine in previous seasons. (*Grade B Evidence*)**

### Summary of evidence

• Repeated vaccination across seasons, including the current season, was consistently more effective than no vaccination in the current season.

• In general, the evidence shows no significant difference or predictable trend in vaccine efficacy or effectiveness between vaccinations in two consecutive seasons compared to vaccination in the current season only.

o Of all the seasons investigated across many studies, only two influenza seasons indicated that VE of vaccination over consecutive seasons was statistically significantly lower than vaccination in the current season only. These notable seasons were influenza A(H3N2) in 2010–2011 ((14)) and influenza A(H3N2) in 2014–2015 ((15)). These findings were not statistically significant in all SRs/MAs that assessed VE in these two seasons; however, a trend towards lower VE for repeated vaccination was consistent for the 2014–2015 season across all studies ((12,14)).

• Evidence on the effects of repeated vaccination over three or more consecutive seasons was limited and is insufficient to draw firm conclusions at this time.

• Given the complex interplay between immune imprinting (such as previous exposures through vaccination and natural infection), circulating virus types and individual characteristics, it is not currently feasible nor warranted to modify existing annual influenza vaccination programs to account for potential negative or positive interference effects related to repeated influenza vaccination across seasons.

A complete review of evidence and full NACI recommendations are published in the new NACI *Recommendation on Repeated Seasonal Influenza Vaccination* ((11)). This guidance aligns with NACI's overarching recommendation for influenza vaccination and standard vaccine administration practices as detailed in the Canadian Immunization Guide and Annual NACI Statement on Seasonal Influenza Vaccine ((1)).

## Conclusion

The body of evidence exploring whether repeated seasonal influenza vaccination can enhance or attenuate influenza vaccine immunogenicity and effectiveness continues to grow. Notably, a recent SR and MA commissioned by the World Health Organization (WHO) Strategic Advisory Group of Experts on Immunization (SAGE) Working Group on Influenza ((16)) examined the available evidence for the potential reduction in VE associated with repeated influenza vaccination. According to the WHO SAGE review, although vaccination in the previous year appears to attenuate VE, vaccination in two consecutive years affords better protection than not being vaccinated. Overall, the WHO SAGE review findings were in alignment with the conclusions of the recent NACI assessment: the effects of vaccination in the previous year were not consistent across seasons and further evaluation and investigation of whether VE is reduced by repeated vaccination would be needed prior to considering an alternative influenza vaccination regimen. New and emerging research priorities identified during NACI's recommendation development process, include the following:

• Effects of long-term repeated influenza vaccination on VE

• Effects of repeated influenza vaccination on VE stratified by age group and vaccine type

• Effects of repeated influenza vaccination on severe influenza-related outcomes, such as hospitalization and death

• Effects of repeated influenza vaccination that accounts for previous influenza exposure through vaccination and/or natural infection

• Immunological mechanisms underlying the effects of repeated influenza vaccination on VE

NACI will continue to monitor the evolving evidence and will update this guidance as needed.

## References

[1] National Advisory Committee on Immunization. Canadian Immunization Guide Chapter on Influenza and Statement on Seasonal Influenza Vaccine for 2022–2023. Ottawa, ON: PHAC; 2023. https://www.canada.ca/en/public-health/services/publications/vaccines-immunization/canadian-immunization-guide-statement-seasonal-influenza-vaccine-2022-2023.html

[2] Hoskins TW, Davies JR, Smith AJ, Miller CL, Allchin A. Assessment of inactivated influenza-A vaccine after three outbreaks of influenza A at Christ’s Hospital. Lancet 1979;1(8106):33–5. 10.1016/S0140-6736(79)90468-9 83475

[3] Sullivan SG, Kelly H. Stratified estimates of influenza vaccine effectiveness by prior vaccination: caution required. Clin Infect Dis 2013 Aug;57(3):474–6. 10.1093/cid/cit255 23619811

[4] Skowronski DM, Chambers C, De Serres G, Sabaiduc S, Winter AL, Dickinson JA, Gubbay JB, Fonseca K, Drews SJ, Charest H, Martineau C, Krajden M, Petric M, Bastien N, Li Y, Smith DJ. Serial Vaccination and the Antigenic Distance Hypothesis: Effects on Influenza Vaccine Effectiveness During A(H3N2) Epidemics in Canada, 2010-2011 to 2014-2015. J Infect Dis 2017;215(7):1059–99. 10.1093/infdis/jix074 28180277 PMC5853783

[5] McLean HQ, Thompson MG, Sundaram ME, Kieke BA, Gaglani M, Murthy K, Piedra PA, Zimmerman RK, Nowalk MP, Raviotta JM, Jackson ML, Jackson L, Ohmit SE, Petrie JG, Monto AS, Meece JK, Thaker SN, Clippard JR, Spencer SM, Fry AM, Belongia EA. Influenza vaccine effectiveness in the United States during 2012-2013: variable protection by age and virus type. J Infect Dis 2015;211(10):1529–40. 10.1093/infdis/jiu647 25406334 PMC4407759

[6] Puig-Barberà J, Burtseva E, Yu H, Cowling BJ, Badur S, Kyncl J, Sominina A. GIHSN. Influenza epidemiology and influenza vaccine effectiveness during the 2014–2015 season: annual report from the Global Influenza Hospital Surveillance Network. BMC Public Health 2016;16(Suppl 1 Suppl 1):757. https://bmcinfectdis.biomedcentral.com/articles/10.1186/s12879-019-4017-0 10.1186/s12889-016-3378-1 PMC5001209 27556802

[7] Thompson MG, Naleway A, Fry AM, Ball S, Spencer SM, Reynolds S, Bozeman S, Levine M, Katz JM, Gaglani M. Effects of Repeated Annual Inactivated Influenza Vaccination among Healthcare Personnel on Serum Hemagglutinin Inhibition Antibody Response to A/Perth/16/2009 (H3N2)-like virus during 2010-11. Vaccine 2016;34(7):981–8. 10.1016/j.vaccine.2015.10.119 26813801 PMC5218812

[8] Smith DJ, Forrest S, Ackley DH, Perelson AS. Variable efficacy of repeated annual influenza vaccination. Proc Natl Acad Sci USA 1999;96(24):14001–6. 10.1073/pnas.96.24.14001 10570188 PMC24180

[9] Ismail SJ, Hardy K, Tunis MC, Young K, Sicard N, Quach C. A framework for the systematic consideration of ethics, equity, feasibility, and acceptability in vaccine program recommendations. Vaccine 2020;38(36):5861–76. 10.1016/j.vaccine.2020.05.051 32532544 PMC7283073

[10] National Advisory Committee on Immunization. Evidence-based recommendations for immunization-Methods of the National Advisory Committee on Immunization. Can Commun Dis Rep. 2009;35(ACS-1):1–10. https://www.canada.ca/content/dam/phac-aspc/migration/phac-aspc/publicat/ccdr-rmtc/09pdf/ccdr-rmtc-vol-35-acs-dcc-1.pdf 19192504

[11] National Advisory Committee on Immunization. Recommendation on Repeated Seasonal Influenza Vaccination. Ottawa, ON: PHAC; 2023. https://www.canada.ca/en/public-health/services/immunization/national-advisory-committee-on-immunization-naci/recommendation-repeated-seasonal-influenza-vaccination.html 10.14745/ccdr.v49i04a02 PMC10826901 38298903

[12] Belongia EA, Skowronski DM, McLean HQ, Chambers C, Sundaram ME, De Serres G. Repeated annual influenza vaccination and vaccine effectiveness: review of evidence. Expert Rev Vaccines 2017;16(7):1–14. 10.1080/14760584.2017.1334554 28562111

[13] Morimoto N, Takeishi K. Change in the efficacy of influenza vaccination after repeated inoculation under antigenic mismatch: A systematic review and meta-analysis. Vaccine 2018;36(7):949–57. 10.1016/j.vaccine.2018.01.023 29373191

[14] Bartoszko JJ, McNamara IF, Aras OA, Hylton DA, Zhang YB, Malhotra D, Hyett SL, Morassut RE, Rudziak P, Loeb M. Does consecutive influenza vaccination reduce protection against influenza: A systematic review and meta-analysis. Vaccine 2018;36(24):3434–44. 10.1016/j.vaccine.2018.04.049 29724509

[15] Ramsay LC, Buchan SA, Stirling RG, Cowling BJ, Feng S, Kwong JC, Warshawsky BF. The impact of repeated vaccination on influenza vaccine effectiveness: a systematic review and meta-analysis. BMC Med 2019;17(1):9. 10.1186/s12916-018-1239-8 30626399 PMC6327561

[16] Jones-Gray E, Robinson EJ, Kucharski AJ, Fox A, Sullivan SG. Does repeated influenza vaccination attenuate effectiveness? A systematic review and meta-analysis. Lancet Respir Med 2023;11(1):27–44. 10.1016/S2213-2600(22)00266-1 36152673 PMC9780123

